# Beta-lactam antibiotics have immunomodulatory effects *in vitro*, including changes consistent with sepsis-induced immunosuppression

**DOI:** 10.1186/s13054-026-06100-y

**Published:** 2026-08-17

**Authors:** Timothy Arthur Chandos Snow, Sunny Charoenpong, Haeun Kim, Keshava Carthigesan, Laura Gallagher, Samer Elkhodair, Roi Avraham, Abhishek Das, Mervyn Singer, David Brealey, Nishkantha Arulkumaran, the University College London Hospitals Critical Care Research Team, Georgia Bercades, Antonio Cesar, Ingrid Hass, Alexandra Zapata Martinez, Gladys Martir, Holly Pan, Walter Pisciotta, Francis Ryckaert, Naveed Saleem, Deborah Smyth, Alessia V. Waller

**Affiliations:** 1 Bloomsbury Institute of Intensive Care Medicine, Division of Medicine, University College London, Gower St, London, WC1E 6BT UK; 2 Centre for Anaesthesia, Critical Care & Pain Medicine, Division of Targeted Intervention, University College London, London, UK; 3 Emergency Department, University College London Hospitals NHS Foundation Trust, London, UK; 4 Department of Immunology and Regenerative Biology, Weizmann Institute of Science, Rehovot, Israel; 5 Division of Infection & Immunity, University College London, London, UK; 6 NIHR UCLH Biomedical Research Centre, University College London Hospitals NHS Foundation Trust, London, UK

**Keywords:** Anti-Bacterial Agents, beta-Lactams, Immunomodulation, Lymphocyte, Monocyte, Sepsis

## Abstract

**Introduction:**

Sepsis is associated with immunosuppression, predisposing patients to secondary infections. Many treatments routinely used for infections have immunomodulatory effects, including antibiotics. We therefore assessed the immunomodulatory effects of beta-lactam antibiotics on monocyte and lymphocyte immunophenotype.

**Methods:**

Peripheral blood mononuclear cells isolated from Emergency Department patients with bacterial infection were incubated with narrow-spectrum (amoxicillin and cefuroxime) or broad-spectrum (piperacillin-Tazobactam and meropenem) beta-lactam antibiotics at low and high concentrations. We compared the effects of antibiotics with and without an additional stimulus, LPS for 24 h or anti-CD3/CD28 beads for 72 h to evaluate the effect on monocyte and lymphocyte phenotype respectively. Using spectral flow cytometry, we evaluated functional markers associated with immune activation and reproducible phenotypes consistent with sepsis-induced immunosuppression.

**Results:**

Beta-lactams, at higher-dose, were associated with increased monocyte CCR2 and decreased CD14 expression. Cefuroxime, meropenem and piperacillin had additional effects, causing a reduced monocyte HLA-DR, NOX-2, CLIP, and NF-κB expression with increased CD80. Beta-lactam exposure was associated with increased CD4^+^ lymphocyte viability, whilst amoxicillin had additional effects including reduced PD-1 expression and proliferation, and increased IL-7R expression. Changes to immune cell phenotype were minimal in CD8^+^ lymphocytes, at lower antibiotic doses, or in unstimulated cells.

**Conclusion:**

Beta-lactam antibiotics have immunomodulatory effects in vitro, including changes consistent with sepsis-induced immunosuppression at clinically relevant doses. Further work is required to determine the mechanisms underpinning these observations and clinical implications, which may have significant clinical implications on the type and duration of antibiotics administered, and highlight the need for therapeutic antibiotic monitoring.

**Supplementary Information:**

The online version contains supplementary material available at 10.1186/s13054-026-06100-y.

## Introduction

Sepsis, the dysregulated host response to infection, is associated with immunosuppression which predisposes patients to secondary infections and increased mortality risk [1, 2]. The cause of sepsis-induced immunosuppression is multifactorial and includes the inadvertent effects of routinely administered drugs [3]. 

Antibiotics, the cornerstone of sepsis management, are known to modulate the immune system [4]. Several classes of antibiotics have immunosuppressive effects, including beta-lactam antibiotics, the most commonly used class for sepsis management [5]. These effects have been characterised predominantly in cell lines and animal models [5], with limited data in critically ill patients [4, 6]. 

**Fig. 1:**
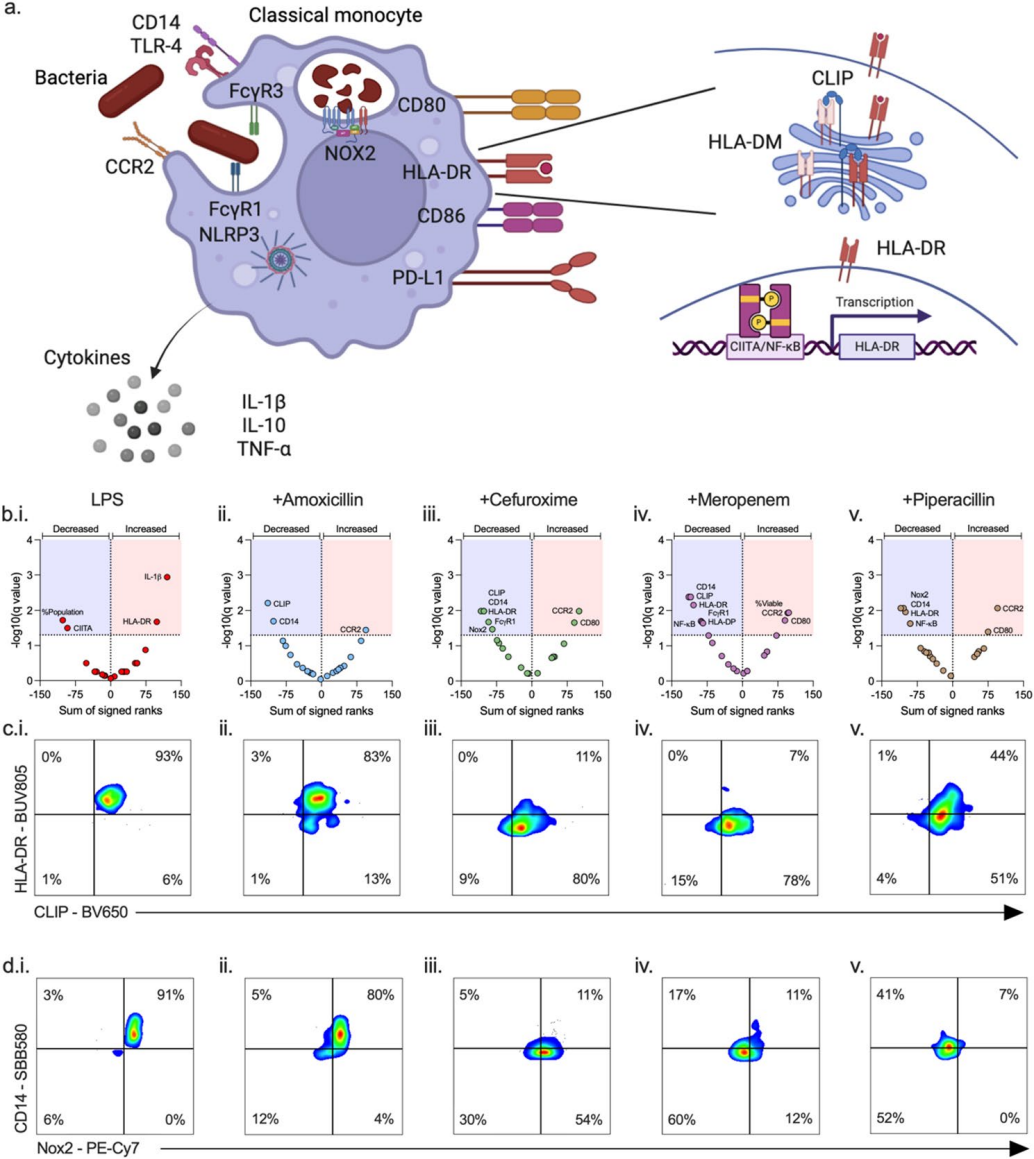
High dose beta-lactam antibiotics exacerbate sepsis-induced impairments in monocyte antigen presentation and phagocytosis pathways ex vivo. PBMCs isolated from patients presenting to the Emergency Department with likely bacterial infection (*n* = 26) were stimulated for 24 h with LPS (100ng/ml, red dots) and co-incubated with either amoxicillin (25 µg/ml, blue shading), cefuroxime (25 µg/ml, green shading), meropenem (60 µg/ml, purple shading), or piperacillin (250 µg/ml, brown shading) and the effect on classical monocyte phenotype assessed. (**a**.) Summary diagram of monocyte markers assessed created in BioRender. (**b.-d**.) Effect on measured monocytes markers of (i.) LPS vs. control, with either (ii.) amoxicillin, (iii.) cefuroxime, (iv.) meropenem, or (v.) piperacillin vs. LPS presented as (**b**.) volcano plots, or representative flow cytometry contour plots of (**c**.) HLA-DR and CLIP, and (**d**.) CD14 and Nox2 expression with gates drawn based on the LPS sample for demonstration. Data analysed using multiple Wilcoxon tests with correction for multiple comparisons, with only those significantly upregulated (red shaded area) or downregulated (blue shaded area) listed

**Fig. 2:**
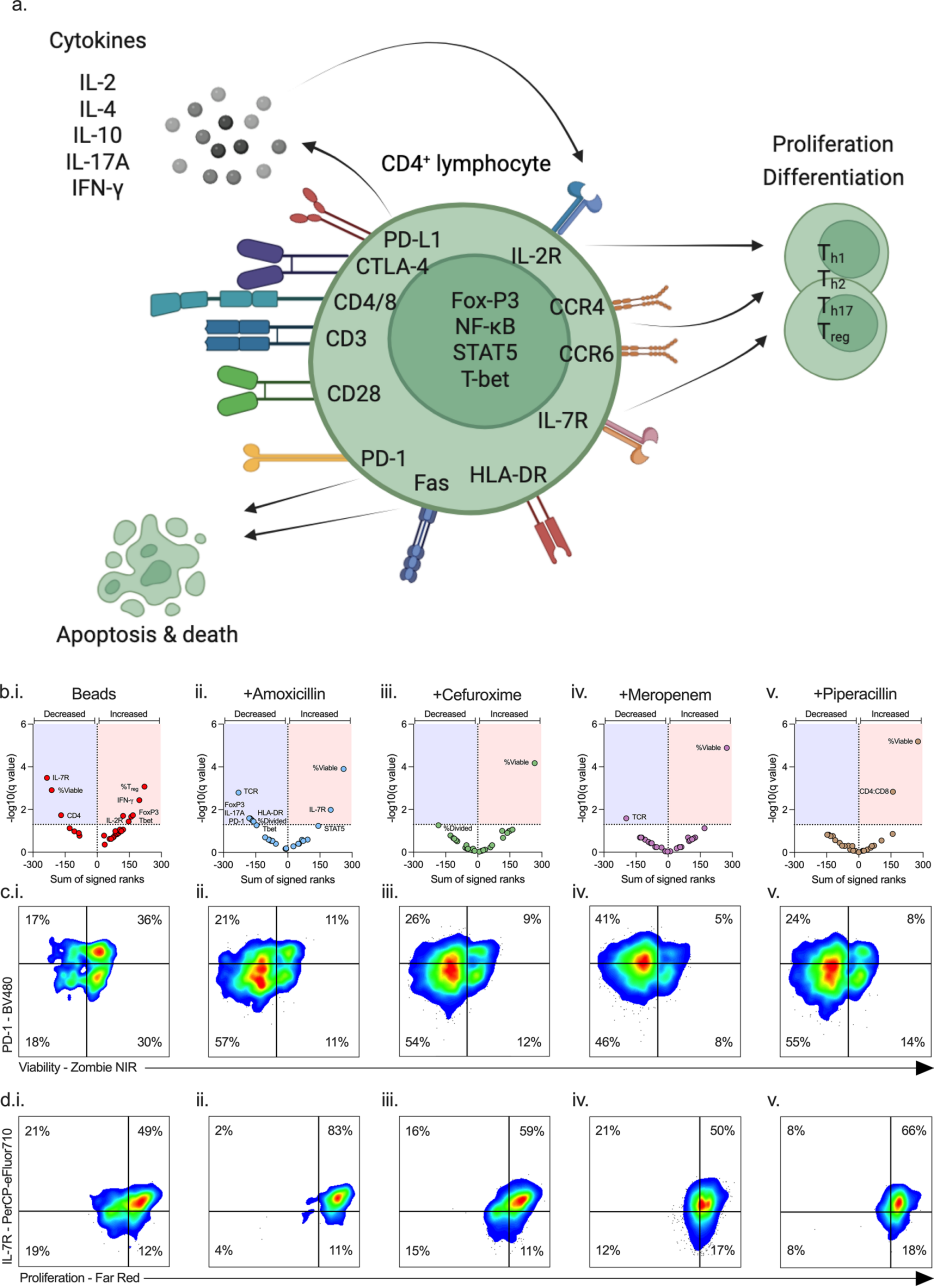
High-dose beta-lactam antibiotics may reduce sepsis-induced lymphopenia in CD4^+^ lymphocytes through reduced activation-associated cell death. PBMCs isolated from patients presenting to the Emergency Department with presumed infection (*n* = 26) were stimulated for 72 h with anti-CD3/CD28 beads (4:1 beads: PBMCs, red dots) with either amoxicillin (25 µg/ml, blue shading), cefuroxime (25 µg/ml, green shading), meropenem (60 µg/ml, purple shading), or piperacillin (250 µg/ml, brown shading) and the effect on CD4^+^ lymphocyte phenotype assessed. (**a**.) Summary diagram of lymphocyte markers assessed created in BioRender. (**b.-d**.) Effect on measured monocytes markers of (i.) Beads vs. control, and either (ii.) amoxicillin, (iii.) cefuroxime, (iv.) meropenem, or (v.) piperacillin vs. beads presented as (**b**.) volcano plots, or representative contour plots of (**c**.) PD-1 and viability, and (**d**.) IL-7R and proliferation with gates drawn based on the Beads sample for demonstration. Data analysed using multiple Wilcoxon tests with correction for multiple comparisons, with only those significantly upregulated (red shaded area) or downregulated (blue shaded area) listed

To that end, we assessed if beta-lactam antibiotics exacerbate features of sepsis-induced immunosuppression. We conducted a prospective study to assess the in vitro effects of narrow-spectrum (amoxicillin and cefuroxime) and broad-spectrum (meropenem and piperacillin) beta-lactam antibiotics on monocyte and lymphocyte phenotype using peripheral blood mononuclear cells (PBMCs) isolated from patients presenting to the Emergency Department (ED) with bacterial infection. Using spectral flow cytometry, we evaluated functional markers associated with phenotypes consistent with sepsis-induced immunosuppression including expression of markers associated with reduced CD4^+^ lymphocyte function and viability, and reduced monocyte HLA-DR expression [1, 2, 7]. (Figures 1a and 2a)

## Methods

Full methods are available in Supplemental material.

Ethical approval was granted by the London – Queen Square Research Ethics Committee for a prospective observational study of patients aged ≥ 18 years who presented to the Emergency Department at University College London (UCL) Hospitals between 1st August 2021 and 26th January 2023 who had blood cultures taken for suspicion of bacterial infection prior to administration of antibiotics. Exclusion criteria included patients with underlying hemato-oncologic diagnoses, patients on chronic immunosuppressive medications, severe anaemia with a contra-indication to blood transfusion, those not expected to survive beyond 24 h of admission, and pregnant women.

PBMCs were isolated, cryopreserved and analysed in batches. PBMCs were incubated with or without two doses of beta-lactam antibiotics (amoxicillin and cefuroxime (both 5 and 25 µg/ml), meropenem (20 and 60 µg/ml), or piperacillin-Tazobactam (50 and 250 µg/ml)) for 24 h to assess monocyte response, or 72 h to assess lymphocyte response. Doses represented clinically relevant lower and higher mean serum concentrations derived from pharmacokinetic data obtained in critically ill patients [8]. 

To assess the effect of antibiotics on stimulated cells, LPS (100ng/ml) was used to assess monocyte immunophenotype, and anti-CD3/CD28 beads (4:1 beads: PBMCs) was used to assess lymphocyte immunophenotype based on previous experiments [6, 9]. 

After incubation, cells were stained for acquisition on an ID7000 spectral cell analyser with panels designed to assess monocyte (Supplemental Table 1) or lymphocyte (Supplemental Table 2) immunophenotype.

## Results

### Patient cohort

26 patients were included; with a median age of 64 (47–75) and 54% were female. Sources of infection included chest (*n* = 12, 46%), genitourinary (*n* = 7, 27%), soft tissue (*n* = 3, 12%), gastrointestinal (*n* = 2, 8%), central nervous system (*n* = 1) and ear, nose and throat (*n* = 1). Inflammatory markers were elevated in keeping with infection; WCC 12.5 (10.4–17.5) x10^6^ cells/ml and CRP 83 (33–176) mg/dL, and NEWS2 (National Early Warning Score 2) score was elevated 4 [2–7]. Two patients were at increased risk of poor outcome from their infection as defined by a qSOFA score ≥2. 69% of patients were admitted to hospital for treatment and in-hospital mortality was 19%. Full details on clinical data are available in Supplemental Table 3. Antibiotic exposure in vitro was associated with few changes to unstimulated monocytes or CD8^+^ lymphocytes, and no changes in CD4^+^ lymphocytes (Supplementary Figs. 1–3).

### Monocyte phenotype

Exposure of PBMCs to LPS for 24 h resulted in an increased expression of classical monocyte HLA-DR and IL-1β, and reduction in CIITA expression and percentage classical monocytes (Fig. 1c.i**.**, Supplemental Fig. 1). Beta-lactams demonstrated an effect on monocyte immunophenotype at the higher clinically relevant concentration compared to LPS-stimulation alone, with a reduction in expression of CD14 and an increase in expression of CCR2 (Fig. 1c.ii-v., 1e.). Broad-spectrum beta-lactams (meropenem and piperacillin) were associated with a reduction in NF-κB (Fig. 1c.iv-v.). Amoxicillin, cefuroxime and meropenem were associated with a reduction in CLIP (Fig. 1c.ii-iv. and 1d). Cefuroxime, meropenem, and piperacillin were associated with a reduction in HLA-DR (Fig. 1c.iii-v., 1d). Cefuroxime and meropenem caused a reduction in FcγR1 (Figure 1iii.-iv.). Cefuroxime and piperacillin caused a reduction in Nox-2 (Fig. 1c.iii, v., 1e.). Additionally, meropenem caused a reduction in HLA-DP expression and an increase in monocyte viability (Fig. 1c.iv.). Changes with lower-dose antibiotics were less evident (Supplemental Fig. 1 and Supplemental Fig. 2).

### Lymphocyte phenotype

Exposure of PBMCs to anti-CD3/CD28 beads resulted in an increase in CD4^+^ lymphocyte %T_regs_, IFN-γ, IL-2R, FoxP3, and Tbet, with a concurrent reduction in the expression of IL-7R, CD4 and %Viable cells (Fig. 2c.i., Supplemental Fig. 2 and Supplemental Fig. 3). Exposure of PBMCs to anti-CD3/CD28 beads resulted in an increase in CD8^+^ lymphocyte HLA-DR, PD-1, IL-4, IL-2R, FoxP3, Fas, Tbet, and %T_c1_ population. There was a concurrent decrease in CD8^+^ lymphocyte IL-7R, STAT5, CCR4, CD3, and %Viable cells (Supplemental Fig. 3 and Supplemental Fig. 4).

Compared to anti-CD3/CD28 bead-stimulation alone, high dose amoxicillin ameliorated changes typically associated with sepsis-induced immunosuppression; including decreased PD-1 expression and increased IL-7R expression and viability of CD4^+^ lymphocytes (Fig. 2.c.ii., d., e.). Expression of CD3, FoxP3 and IL-17A were reduced (Fig. 2.c.ii.). Few changes were associated with low-dose amoxicillin (Supplemental Fig. 2 and Supplemental Fig. 3).

Cefuroxime, meropenem, and piperacillin were associated with an increase in CD4^+^ lymphocyte viability compared to anti-CD3/CD28 bead stimulation alone (Fig. 2c.iii-v. and d.). Additionally, cefuroxime increased the CD4:CD8 ratio and decrease in %divided CD4^+^ lymphocytes (Fig. 2c.iii.); and piperacillin was associated with a reduction in PD-1, IL-2R and %divided cells at lower dose (Supplemental Fig. 2 and Supplemental Fig. 3).

Beta-lactam antibiotics were not associated with any changes to CD8^+^ lymphocytes (Supplemental Fig. 4).

### Released cytokines

Following 24 h stimulation with LPS alone, levels of IL-1β and IL-10 were increased in the supernatant. This was unaffected by beta-lactam antibiotics. Following 72 h stimulation with anti-CD3/CD28 beads alone, levels of IFN-γ but not IL-10 were elevated in the supernatant. IFN-γ was unaffected by beta-lactam antibiotics. Co-stimulation with anti-CD3/CD28 beads and high dose cefuroxime, meropenem, and piperacillin were associated with an increase in supernatant IL-10 (Supplemental Fig. 5).

## Discussion

We demonstrate, for the first time, that routinely used beta-lactam antibiotics modulate monocyte and CD4^+^ lymphocyte phenotype in vitro at clinically relevant doses in patients with an acute infection. Amoxicillin predominantly affects stimulated CD4^+^ lymphocytes, whereas cefuroxime, meropenem, and piperacillin-tazobactam predominantly affect stimulated monocytes. Excluding amoxicillin, all beta-lactam antibiotics increase released IL-10 following 72 h stimulation. No clear differences between broad and narrow-spectrum beta-lactam antibiotics were demonstrated but the effects were predominantly evident at higher compared to lower clinically relevant doses, consistent with a dose-dependent effect.

Cefuroxime, piperacillin, and meropenem were associated with changes primarily to monocyte phenotype and were associated with features of sepsis-induced immunosuppression [1, 7], including a reduction in classical monocyte HLA-DR. Monocyte HLA-DR is regulated both at the transcriptional and post-transcriptional levels, with primarily CIITA but also NF-κB as the main transcriptional regulators [10, 11]. Whilst CIITA expression was unaffected by beta-lactams, meropenem and piperacillin were associated with reduced NF-κB expression. Cefuroxime and meropenem also reduced expression of CLIP, a key post-translational regulator of HLA-DR [11, 12]. 

All beta-lactam antibiotics were associated with an increase in CD4^+^ lymphocyte viability and reduction in proliferation. In non-immune cells, beta-lactams induce apoptosis mediated by direct DNA damage [13]. Increased viability in CD4^+^ lymphocytes is associated with a reduction in expression of PD-1 (by amoxicillin and piperacillin), a receptor associated with both increased cell anergy and death. Additionally, improved CD4^+^ lymphocyte viability associated with amoxicillin could be mediated via a reduction in activation-associated cell death as it was associated with a reduction in expression of markers associated with CD4^+^ lymphocyte activation. Although in allergy models, amoxicillin binds to the T-cell receptor causing activation, in those without a hypersensitivity reaction to amoxicillin, receptor binding may result in antagonism [14]. Previous data has demonstrated conflicting effects of beta-lactams on proliferation with either no effect [15], or a reduction [16], consistent with our findings.

Excluding amoxicillin, all other beta-lactams caused an increase in lymphocyte-released IL-10 in the supernatant at 72 h. The reported effects of beta-lactams IL-10 release are conflicting; including an increased serum IL-10 by carbapenems and amoxicillin in a 5 day rat model of ear infection [17], but reduced serum IL-10 after 7 days in patients with S. *aureus* bacteraemia [18], and in a rat model of S. *aureus* osteomyelitis [19]. Lymphocyte IL-10 production is regulated by multiple pathways, including the T-cell receptor (CD3), CD28, and NF-κB [20]. Whilst we measured the relative expression of these markers, beta-lactams either reduced expression or had no effect suggesting alternative mechanisms.

Most of the immunosuppressive effects demonstrated occurred at higher concentrations observed in clinical practice. The use of therapeutic drug monitoring (TDM) has been advocated in the critically ill to ensure serum levels remain above the minimum inhibitory concentration to reduce the risk of treatment failure. Our data supports the implementation of TDM to ensure serum concentrations are not significantly higher than the desired therapeutic concentration to minimise inadvertent toxicity.

We acknowledge some limitations. We conducted all in vitro work using PBMCs isolated from patients in the emergency department with infection but without acute organ dysfunction, as patients being admitted to the ICU have invariably been exposed to several medications including antibiotics. Data on clinical surrogates of immunosuppression including secondary infections or viral reactivation were unavailable. We chose a single dose of LPS and anti-CD3/CD28 beads for cell stimulation; the immune effects may differ with other doses or stimuli. We assessed relative expression of markers associated with common immune cell functions, but due to limitations in cell numbers did not assess function beyond cytokine release or lymphocyte proliferation and differentiation. There are no specific features of immune dysfunction which are unique to sepsis, changes in most of the markers assessed likely represent a continuum of infection severity [7, 10]. Nonetheless, a number of these features have been used as therapeutic or theragnostic targets in sepsis. For example, monocyte HLA-DR was used to stratify patients to treatment with IFN-γ in the ImmunoSep trial [21]. 

We provide novel and thought-provoking data on the immunomodulatory effects of beta-lactam antibiotics. At clinically relevant doses, beta-lactam antibiotics have immunomodulatory effects in vitro on immune cell function, including changes consistent with features of sepsis-induced immunosuppression. The mechanism underpinning our findings likely relates to the effect of immune cells reacting to the antibiotic in the presence of an infectious stimulus. Further work is required to understand the mechanisms, functional consequences, and clinical implications including the role of TDM. Our work is thus exploratory and provides conceptual consideration for clinicians, immunologists, microbiologists and scientists.

## Electronic Supplementary Material

Supplementary Material 1

## Data Availability

The raw data supporting the conclusions of this article will be made available by the authors, without undue reservation.
