# Supplementary material for: Beta-lactam antibiotics have immunomodulatory effects in vitro, including changes consistent with sepsis-induced immunosuppression
Source: Crit Care. 2026 Aug 17;30:424. doi: 10.1186/s13054-026-06100-y (PMC13483138; doi:10.1186/s13054-026-06100-y)
Supplement: Supplementary file 1 [file 13054_2026_6100_MOESM1_ESM.docx]

Supplementary material

# Supplementary Methods

## Ethics

Ethical approval for this study was granted by the London – Queen Square Research Ethics Committee (REC reference 20/LO/1024), as a parallel study investigating biomarkers in sepsis.

## Study participants

We conducted a prospective observational study of patients aged ≥18 years who presented to the Emergency Department at University College London (UCL) Hospitals between 1^st^ August 2021 and 26^th^ January 2023 who had blood cultures taken for suspicion of bacterial infection. Exclusion criteria included patients with underlying hemato-oncologic diagnoses, patients on chronic immunosuppressive medications, severe anaemia with a contra-indication to blood transfusion, those not expected to survive beyond 24 hours of admission, and pregnant women. Patient demographics, clinical data (physiology, diagnoses, etc.), laboratory data, and clinical outcomes were recorded from electronic healthcare records until death or discharge.

Based on our previous data in surgical patients demonstrating a monocyte HLA-DR median fluorescence intensity (MFI) of 5,000 ± 1,250,(9) with a power of 80%, and alpha of 0.05, a sample size of ≥ 25 patients per group was required to detect a statistically significant difference of 20% between groups.

## Sample Processing

Patients were recruited and blood samples obtained prior to administration of antibiotics. Venesection was performed and 8mls of blood drawn into a cell preparation tube with sodium heparin (CPT^TM^, Becton Dickinson, Wokingham, UK) vacutainer and processed within 1 hour. The CPT^TM^ vacutainers were centrifuged at 1500g for 15 minutes at room temperature and the peripheral blood mononuclear cell (PBMC) layer extracted, washed twice in 2mls of phosphate buffered saline (PBS) and resuspended in freezing media (foetal bovine serum (FBS, Gibco, Thermo Fisher Scientific, Horsham, UK) supplemented with 10% dimethyl sulfoxide (Merck Life Sciences, Gillingham, Dorset)) and frozen to -80^o^C using an isopropyl alcohol system (Mr Frosty^TM^, Thermo Fisher Scientific). Samples were transferred to liquid nitrogen within 48 hours for long-term storage.

PBMCs were defrosted in batches by resuspension in cell culture media (RPMI Glutamax, Gibco) with 10% FBS (Gibco), washed once in media, counted (Countess 3 Automated cell counter, Thermo Fisher Scientific), and diluted to a concentration of 1x10^6^ cells/ml.

## *In vitro* stimulation

PBMCs were plated into 96-well plates and incubated at 37^o^C, 5% CO_2_ with or without beta-lactam antibiotics for 24 hours to assess monocyte response, or 72 hours to assess lymphocyte response. Amoxicillin (Wockhardt, Wrexham, UK), cefuroxime (Flynn Pharma, Stevenage, UK), meropenem (Milpharm, Ruislip, UK), and piperacillin- Tazobactam (Fresenius Kabi, Runcorn UK) were first resuspended in distilled water as per manufacturer’s instructions before being diluted to working stock concentration in PBS for addition to relevant wells. Final concentrations of 5 and 25 µg/ml for amoxicillin and cefuroxime, 20 and 60 µg/ml for meropenem, and 50 and 250 µg/ml for piperacillin were chosen to represent clinically relevant lower and higher mean serum concentrations respectively based on data from pharmacokinetic data obtained in critically ill patients.(8) Culture medium with relevant antibiotic concentrations were replaced every 24 hours to account for spontaneous degradation of antibiotics.

To assess the effect of antibiotics on stimulated cells, LPS (Sigma-Aldrich, Gillingham, UK) at a concentration of 100ng/ml was used to assess monocyte immunophenotype, and anti-CD3/CD28 beads (Miltenyi Biotec, Woking, UK) at a concentration of 4:1 (beads: PBMCs) was used to assess lymphocyte immunophenotype. Doses were based on previous experiments.(6) Following stimulation, 96-well plates were centrifuged at 400g for 5 minutes at room temperature the supernatant removed and stored at -80^o^C for subsequent cytokine measurement using ELISA.

## Flow cytometry

To assess effect of antibiotics on markers associated with monocyte function, PBMCs were resuspended in cell staining buffer (BioLegend, London, UK) and incubated with fluorochrome-labelled antibodies to the following markers (CD11b, CD14, CD16 (FcγR3), CD64 (FcγR1), CD74 (CLIP), CD80, CD86, CD274 (PD-L1), CD284 (TLR4), and HLA-DM/DP/DR) and viability stain (Live/Dead). Following use of the Tru-nuclear fixation/permeabilization kit (BioLegend) as per manufacturer recommendations, fluorochrome-labelled antibodies to the following intracellular cytokines (IL-1β, IL-10, IFN-γ, and TNF-α), proteins (NLRP3 and NOX-2), and transcription factors (NF-κB, and CIITA) were added. Details of products and concentrations used are listed in **Supplemental Table 1**.

For lymphocyte characterisation, PBMCs were resuspended in cell staining buffer (BioLegend) with fluorochrome-labelled antibodies to the following cell surface markers (CD3, CD4, CD8, CD19, CD25 (IL-2R), CD28, CD95 (Fas), CD127 (IL-7R), CD152 (CTLA-4), CD194 (CCR4), CD196 (CCR6), CD274 (PD-L1), CD279 (PD-1), and HLA-DR) and viability stain (Live/Dead). After use of the Tru-nuclear fixation/permeabilization kit (BioLegend) as per manufacturer recommendations, fluorochrome-labelled antibodies to the following intracellular cytokines (IL-2, IL-4, IL-10, IL-17A, and IFN-γ), and transcription factors (NF-κB, Fox-P3, STAT5, and T-bet) were added. Details of products and concentrations used are listed in **Supplemental Table 2**.

Cells were acquired on an ID7000 spectral cell analyser (Sony Biotechnology Inc, Weybridge, UK) and analysed using ID7000 software (version 1.2, Sony). Alignment check beads (Sony) were used prior to running each experiment and spectral references for each fluorochrome added using single stain labelled heat-killed cells (60^o^C for 10minutes, live/dead stain) or compensation beads (Thermo Fisher Scientific, all other markers) with appropriate negative controls. FMO (fluorescence minus one) samples were used to identify cell populations. The stopping gate was set at 10,000 events for either CD14^++^CD16^-^ monocytes or CD4^+^ lymphocytes as per the example gating strategy shown in **Supplemental Figures 1** and **3**.

## Cytokine ELISA

Supernatant IFN-γ, IL-1β, IL-10, and TNF-α were measured using Duoset ELISA kits (R&D Systems, Abingdon, UK) as per manufacturer instructions. Samples were diluted 1:2 in reagent dilutant and optical densities acquired on a SPECTROstar Nano microplate reader (BMG Labtech, Aylesbury, UK).

## Statistics

Clinical and demographic data are presented either as median (inter-quartile range) or number (percentage).

Multivariate analysis was performed and a p-value corrected for multiple comparisons (-log10) determined using a False Discovery Rate (FDR) of 5% using the two-stage step-up method of Benjamini, Krieger and Yekutieli. Data are presented volcano plots and compare effects of antibiotics alone with unstimulated cells, effect of LPS or anti-CD3/CD28 bead stimulation with unstimulated cells, or the effect of antibiotic and LPS or anti-CD3/CD28 co-stimulated cells with LPS or anti-CD3/CD28 bead stimulation alone.

Flow cytometry data are additionally presented as either median fluorescence intensity (MFI; arbitrary units), percentage positive cells, percentage of parent population, or ratio as appropriate and compared using Friedmans test with Dunns uncorrected test for multiple comparisons.

Statistical analyses were performed and graphs constructed using GraphPad Prism software (version 10, GraphPad, San Diego, CA).

# Supplementary Tables

| Function | Marker type | Cell Marker | Fluorochrome | Cat no | Species | Isotope | Dilution |
| --- | --- | --- | --- | --- | --- | --- | --- |
| Gating | Cell surface | CD11b | SBUV445 | BR MCA711 | Mouse | IgG2b | 1:250 |
|  | Cell surface | CD14 | SBB580 | BR MCA1568 | Mouse | IgG2a | 1:250 |
|  | Cell surface | CD16 (FcγR3) | SB702 | TF 67-0168-42 | Mouse | IgG1 | 1:250 |
|  | Cell surface | HLA-DR | BUV805 | BD 748338 | Mouse | IgG2a | 1:250 |
| Chemokine receptor | Cell surface | CD192 (CCR2) | BUV395 | BD 747854 | Mouse | IgG2a | 1:250 |
| Phagocytosis | Cell surface | CD64 (FcγR1) | BUV737 | BD 612776 | Mouse | IgG1 | 1:250 |
|  | Intracellular protein | NOX-2 | PE-Cy7 | BT NBP1-41012 | Mouse | IgG1 | 1:100 |
| Antigen presentation | Cell surface | CD74 (CLIP) | BV650 | BD 743734 | Mouse | IgG1 | 1:250 |
|  | Cell surface | CD80 | BV480 | BD 751735 | Mouse | IgG1 | 1:250 |
|  | Cell surface | CD86 | BUV496 | BD 749895 | Mouse | IgG1 | 1:250 |
|  | Cell surface | HLA-DM | APC | MB 130-124-252 | Human | IgG1 | 1:250 |
|  | Cell surface | HLA-DP | RB780 | BD 755757 | Mouse | IgG1 | 1:250 |
|  | Transcription factor | CIITA | DY680 | BT NBP2-59072FR | Rabbit | IgG | 1:100 |
| T-cell suppression | Cell surface | CD274 (PD-L1) | RB545 | BD 756359 | Mouse | IgG1 | 1:250 |
| Toll-like receptor | Cell surface | CD284 (TLR4) | SB600 | TF 63-9917-42 | Mouse | IgG2a | 1:250 |
| Viability | Viability | Live/Dead | Zombie NIR | BL 423106 | - | - | 1:1000 |
| Cytokine | Intracellular cytokine | IL-1β | AF750 | BT FAB10349S | Human | IgG1 | 1:100 |
|  | Intracellular cytokine | IL-10 | BB700 | BD 566567 | Rat | IgG2a | 1:100 |
|  | Intracellular cytokine | IFN-γ | BV750 | BD 566357 | Mouse | IgG1 | 1:100 |
|  | Intracellular cytokine | TNF-α | BV785 | BL 502948 | Mouse | IgG1 | 1:100 |
| Activation | Intracellular protein | NF-κb p65 | PE-CF594 | BD 565447 | Mouse | IgG2b | 1:100 |
| Inflammasome | Intracellular protein | NLRP3 | AF405 | BT IC7578V | Rat | IgG2a | 1:100 |

### Supplemental Table 1: Flow cytometry fluorochromes used for monocyte analysis.

Abbreviations: APC: Allophycocyanin, AF: Alexa Fluor, BD: Beckton Dickinson, BL: Biolegend, BR: BioRad, BT: BioTechne, BUV: Brilliant ultraviolet, BV: Brilliant violet, CCR: C-C motif chemokine receptor, CD: cluster of differentiation, CF: Cyanine-based fluorescent dye, CLIP: Class II-associated invariant chain peptide, CIITA: Class II major histocompatibility complex transactivator, Cy: Cyanine, DY: DyLight, FcγR: Fragment receptor gamma receptor, HLA: Human leukocyte antigen, IFN: Interferon, IL: Interleukin, L/D: Live/Dead, MB: Miltenyi Biotec, NF-κB: Nuclear Factor Kappa B, NIR: Near-infrared, NLRP3: NLR family pyrin domain containing 3; NOX-2: NADPH oxidase 2; PD-L1: Programmed death-ligand 1, PE: Phycoerythrin, RB: RealBlue, SBV: StarBright violet, SBUV: StarBright ultraviolet, TF: Thermo Fisher, TLR: Toll-like receptor, UV: Ultraviolet.

| **Function** | **Marker type** | **Cell Marker** | **Fluorochrome** | **Cat no** | **Species** | **Isotope** | **Dilution** |
| --- | --- | --- | --- | --- | --- | --- | --- |
| **Cell identification & activation** | Cell surface | CD3 | SBUV445 | BR MCA463 | Mouse | IgG1 | 1:250 |
|  | Cell surface | CD4 | BUV805 | TF 368-0047-42 | Mouse | IgG1, k | 1:250 |
|  | Cell surface | CD8 | APC-Fire 750 | BL 301066 | Mouse | IgG1, k | 1:250 |
|  | Cell surface | CD19 | BUV395 | TF 363-0198-42 | Mouse | IgG1, k | 1:250 |
| **Proliferation** | Proliferation | CellTrace | FarRed | TF C34564 | - | - | 1:1000 |
|  | Cell surface | IL-7R (CD127) | PerCP-eFluor 710 | TF 46-1278-42 | Mouse | IgG1, k | 1:250 |
| **Differentiation** | Cell surface | IL-2R (CD25) | SBV570 | BR MCA2127 | Mouse | IgG1 | 1:250 |
|  | Cell surface | CCR4 (CD194) | BUV563 | BD 752566 | Mouse | IgG1, k | 1:250 |
|  | Cell surface | CCR6 (CD196) | BV786 | BD 563704 | Mouse | IgG1, k | 1:250 |
|  | Intracellular cytokine | IL-2 | BV650 | BL 500334 | Rat | IgG2a, k | 1:100 |
|  | Intracellular cytokine | IL-4 | PE-Cy7 | BD 560672 | Mouse | IgG1, k | 1:100 |
|  | Intracellular cytokine | IL-17A | APC-R700 | BD 565163 | Mouse | IgG1, k | 1:100 |
|  | Intracellular protein | STAT5 | RB780 | BD 568759 | Mouse | IgG1, k | 1:100 |
|  | Transcription factor | Fox-p3 | PE-Cy5 | TF 15-4776-42 | Rat | IgG2a, k | 1:100 |
|  | Transcription factor | T-bet | BV605 | BL 644817 | Mouse | IgG1, k | 1:100 |
| **Activation** | Cell surface | CD28 | BUV496 | BD 741168 | Mouse | IgG1, k | 1:250 |
|  | Cell surface | HLA-DR | BV711 | BD 563696 | Mouse | IgG2a, k | 1:250 |
|  | Intracellular protein | NF-κB | PE-CF594 | BD 565447 | Mouse | IgG2b, k | 1:100 |
| **Suppression** | Cell surface | CTLA-4 (CD152) | AF532 | BT NBP2-50286 | Mouse | IgG1, k | 1:250 |
|  | Cell surface | PD-L1 (CD274) | FITC | BL 393606 | Mouse | IgG1, k | 1:250 |
| **Cell death** | Cell surface | Fas (CD95) | BUV615 | BD 752346 | Mouse | IgG1, k | 1:250 |
|  | Cell surface | PD-1 (CD279) | BV480 | BD 566112 | Mouse | IgG1, k | 1:250 |
|  | Viability | Live/Dead | Zombie NIR | BL 423106 | - | - | 1:1000 |
| **Cytokine release** | Intracellular cytokine | IFN-γ | BV750 | BD 566357 | Mouse | IgG1, k | 1:100 |
|  | Intracellular cytokine | IL-10 | BUV737 | TF 367-7108-42 | Rat | IgG1, k | 1:100 |

### Supplemental Table 2: Flow cytometry fluorochromes used for lymphocyte analysis.

Abbreviations: APC: Allophycocyanin, AF: Alexa Fluor, BD: Beckton Dickinson, BL: Biolegend, BR: BioRad, BT: BioTechne, BUV: Brilliant ultraviolet, BV: Brilliant violet, CCR: C-C motif chemokine receptor, CD: cluster of differentiation, CF: Cyanine-based fluorescent dye, CTLA-4: Cytotoxic T-lymphocyte associated protein-4, Cy: Cyanine, FITC: Fluorescein isothiocyanate, Fox-P3: Forkhead box P3, IFN: Interferon, IL: Interleukin, L/D: Live/Dead, MB: Miltenyi Biotec, NF-κB: Nuclear Factor Kappa B, NIR: Near-infrared, PD-1: Programmed death receptor 1 PD-L1: Programmed death-ligand 1, PE: Phycoerythrin, PerCP: Peridinin-chlorophyll-protein, RB: RealBlue, SBV: StarBright violet, SBUV: StarBright ultraviolet, STAT5: Signal transducer and activator of transcription 5, T-bet: T-box transcription factor TBX21, TF: Thermo Fisher, UV: Ultraviolet.

| Parameter | | Value (n = 26) |
| --- | --- | --- |
| Age (years) | | 64 (47 – 75) |
| Sex (female) | | 14 (54%) |
| Co-morbidities | | |
|  | COPD | 4 (15%) |
|  | Diabetes | 5 (19%) |
|  | Heart failure | 1 (4%) |
|  | Ischemic heart disease | 2 (8%) |
| Source of infection | | |
|  | Chest   - LRTI - IECOPD | 12 (46%)   - 8 - 4 |
|  | Central nervous system   - Meningoencephalitis | 1 (4%)   - 1 |
|  | Ear, Nose and Throat   - Tonsilitis | 1 (4%)   - 1 |
|  | Gastrointestinal   - Terminal ileitis - Duodenal perforation | 2 (8%)   - 1 - 1 |
|  | Genitourinary   - UTI - Pyelonephritis - Orchitis - Infected uterine fibroid | 7 (27%)   - 4 - 1 - 1 - 1 |
|  | Soft tissue   - Cellulitis - Perianal abscess - Mastitis | 3 (12%)   - 1 - 1 - 1 |
| Clinical parameters | | |
|  | Temperature (^o^C) | 37.7 (37.0 – 38.6) |
|  | Respiratory rate | 20 (18 – 26) |
|  | Systolic blood pressure (mmHg) | 126 (109 – 139) |
|  | Heart rate | 112 (92 – 139) |
|  | NEWS2 score | 4 (2 – 7) |
|  | qSOFA | 1 (0 – 1) |
| Laboratory values | | |
|  | WBC (x10^6^/mL) | 12.5 (10.4 – 17.5) |
|  | Neutrophils (x10^6^/mL) | 9.6 (6.5 – 14.0) |
|  | Lymphocytes (x10^6^/mL) | 1.55 (0.82 – 1.97) |
|  | Monocytes (x10^6^/mL) | 0.92 (0.67 – 1.19) |
|  | CRP (mg/l) | 83 (33 – 176) |
|  | Lactate (mmol/l) | 1.0 (0.9 – 1.6) |
| Positive culture results | | |
|  | Blood culture | 0 |
|  | COVID-19/ Flu A and B | 0 |
|  | Urine culture   - E *coli* - K *pneumoniae* | 3 (12%)   - 2 - 1 |
|  | Sputum culture   - H *influenzae* | 1 (4%)   - 1 |
| Outcomes | | |
|  | Hospital admission | 18 (69%) |
|  | ICU admission | 0 |
|  | Mortality | 5 (19%) |

Supplemental Table 3: Clinical data of included patients.

Abbreviations: COPD: Chronic obstructive airway disease, CRP: C-reactive protein, IECOPD: infective exacerbation of COPD, LRTI: Lower respiratory tract infection, NEWS2: National early warning score -2, qSOFA: Quick sequential organ failure assessment, UTI: Urinary tract infection, WBC: White blood cell count

# Supplementary Figures

### Supplemental Figure 1: Effect of antibiotics on monocyte immunophenotype.

PBMCs isolated from patients presenting to the Emergency Department with presumed infection (n=26) were stimulated for 24 hours with amoxicillin (blue dot fill), cefuroxime (green dot fill), meropenem (purple dot fill), or piperacillin (brown dot fill) and the effect on classical monocyte immunophenotype assessed. (a.) Gating strategy. (b.) Effect of high dose antibiotics compared to unstimulated monocytes, and effect of co-stimulation with LPS (100ng/ml) and (c.) low-dose or (d.) high-dose antibiotics compared to monocytes stimulated with LPS alone, presented as volcano plots. Data analysed using multiple Wilcoxon tests; dots represent individual markers of immunophenotype with those listed in the red shaded area significantly upregulated following adjustment for multiple corrections whilst those below the red shaded area (small font in *italics*) were significantly upregulated prior to correction. Those in the blue area were significantly downregulated following adjustment and those below prior to adjustment. (e.-f.) Representative dot plots of effect of high dose antibiotics on individual immunophenotype markers including those associated with (e.) antigen presentation (i. HLA-DR, ii. CD80, iii. CLIP, and iv. CIITA), and phagocytosis (v. CD14, vi. Nox2), (f.) Chemotaxis (i. CCR2), activation (ii. NF-κB), and percentage of total monocyte population for (iii.) classical, (iv.) intermediate, and (v.) non-classical monocytes. Dots represent individual patients, horizontal bar the median, whisker the inter-quartile range and data analysed using Friedman test with Dunns uncorrected test for multiple comparisons.

### Supplemental Figure 2: Heatmap summary of effect of antibiotics on monocyte and lymphocyte immunophenotype.

PBMCs isolated from patients presenting to the Emergency Department with presumed infection (n=26) were incubated with amoxicillin, cefuroxime, meropenem, or piperacillin for 24 hours with or without LPS (a. monocytes) or for 72 hours with or without anti-CD3/CD28 beads (b. CD4^+^ and c. CD8^+^ lymphocytes) and the effect on immunophenotype assessed. Data displayed as a heatmap summary of sum of signed ranks calculated using multiple Wilcoxon tests where red signifies an increase and blue a decrease compared to relevant control: antibiotics without additional co-stimulation were compared with an unstimulated control as was LPS/ beads alone, co-stimulated antibiotics and LPS/ beads compared with LPS/ beads alone. Star symbol denotes significant changes. Different doses of each antibiotic used displayed below each graph.

### Supplemental Figure 3: Effect of antibiotics on CD4^+^ lymphocyte immunophenotype.

PBMCs isolated from patients presenting to the Emergency Department with presumed infection (n=26) were stimulated for 24 hours with amoxicillin (blue dot fill), cefuroxime (green dot fill), meropenem (purple dot fill), or piperacillin (brown dot fill) and the effect on CD4^+^ lymphocyte immunophenotype assessed. (a.) Gating stretgy. (b.) Effect of high dose antibiotics compared to unstimulated CD4^+^ lymphocytes, and effect of co-stimulation with anti-CD3/CD28 beads (4:1 bead: PBMCs) and (c.) low-dose or (d.) high-dose antibiotics on CD4^+^ lymphocytes stimulated with anti-CD3/CD28 beads alone, presented as volcano plots. Data analysed using multiple Wilcoxon tests; dots represent individual markers of immunophenotype with those listed in the red shaded area significantly upregulated following adjustment for multiple corrections whilst those below the red shaded area (small font in *italics*) were significantly upregulated prior to correction. Those in the blue area were significantly downregulated following adjustment and those below prior to adjustment. (e.-f.) Representative dot plots of effect of high dose antibiotics on individual immunophenotype markers including those associated with (e.) activation (i. T-cell receptor/CD3), cell death (ii. PD-1, iii. viability), and proliferation (iv. IL-7R, v. %Divided cells), and (f.) percentage of (i.) T_h1_, (ii.) T_h2_, (iii.) T_h17_, and (iv.) T_reg_ populations. Dots represent individual patients, horizontal bar the median, whisker the inter-quartile range and data analysed using Friedman test with Dunns uncorrected test for multiple comparisons.

### Supplemental Figure 4: Effect of antibiotics on CD8^+^ lymphocyte immunophenotype.

PBMCs isolated from patients presenting to the Emergency Department with presumed infection (n=26) were stimulated for 24 hours with amoxicillin (blue dot fill), cefuroxime (green dot fill), meropenem (purple dot fill), or piperacillin (brown dot fill) and the effect on CD8^+^ lymphocyte immunophenotype assessed. (a.) Effect of high dose antibiotics compared to unstimulated CD8^+^ lymphocytes, and effect of co-stimulation with anti-CD3/CD28 beads (4:1 bead: PBMCs) and (b.) low-dose or (c.) high-dose antibiotics on CD8^+^ lymphocytes stimulated with anti-CD3/CD28 beads alone, presented as volcano plots. Data analysed using multiple Wilcoxon tests; dots represent individual markers of immunophenotype with those listed in the red shaded area significantly upregulated following adjustment for multiple corrections whilst those below the red shaded area (small font in *italics*) were significantly upregulated prior to correction. Those in the blue area were significantly downregulated following adjustment and those below prior to adjustment. (d.) Representative dot plots of effect of high dose antibiotics on percentage of (i.) T_c1_, (ii.) T_c2_, and (iii.) T_c17_ populations. Dots represent individual patients, horizontal bar the median, whisker the inter-quartile range and data analysed using Friedman test with Dunns uncorrected test for multiple comparisons.

### Supplemental Figure 5: Effect of antibiotics on released supernatant cytokines

PBMCs isolated from patients presenting to the Emergency Department with presumed infection (n=26) were incubated with amoxicillin (blue dot fill), cefuroxime (green dot fill), meropenem (purple dot fill), or piperacillin (brown dot fill) for 24 hours (a. monocytes) with or without LPS (monocytes) or for 72 hours with or without anti-CD3/CD28 beads (b. lymphocytes) and the effect on released supernatant cytokine measured. Relevant monocytes cytokines included (a.i.) IL-1β, (a.ii.) IL-10 and (a.iii.) TNF-α. Relevant lymphocyte cytokines included (b.i.) IFN-γ and (b.ii.) IL-10. Data displayed as dot plots where dots represent individual patients, horizontal bar the median, whisker the inter-quartile range and data analysed using Friedman test with Dunns uncorrected test for multiple comparisons.
